# Beyond a gatekeeper: the non-classical signaling role of STRA6 in driving endothelial senescence and atherosclerosis

**DOI:** 10.3389/fimmu.2026.1738384

**Published:** 2026-02-17

**Authors:** Yong Yuan, Jia Wang, Yunfang Zhang, Yan Yu, Weicheng Shi, Sulian Chen, Ye Kuang, Lei Feng

**Affiliations:** Department of Clinical Laboratory, Yan’an Hospital Affiliated to Kunming Medical University, Kunming, China

**Keywords:** atherosclerosis, endothelial senescence, metabolic disease, RBP4, STRA6

## Abstract

Endothelial cell (EC) senescence is a fundamental driver of atherosclerosis. This review posits that the primary pathogenic role of Stimulated by Retinoic Acid 6 (STRA6) in the endothelium is not its canonical vitamin A transport but its non-classical function as a signaling receptor for its ligand retinol-binding protein 4 (RBP4). In metabolic diseases such as obesity and type 2 diabetes, elevated RBP4 levels engage endothelial STRA6, initiating a signaling cascade independent of retinol nuclear activity. This process begins with STRA6 activation of the Janus kinase/signal transducer and activator of transcription (JAK/STAT) pathway. The signal is then amplified via crosstalk with the NLRP3 inflammasome, promoting the secretion of pro-senescent cytokines like Interleukin-1β (IL-1β) and establishing a senescence-associated secretory phenotype (SASP). This pro-inflammatory microenvironment subsequently triggers a persistent DNA damage response (DDR), leading to p53/p21-mediated cell cycle arrest and establishing the full senescent phenotype. This perspective reframes STRA6 as a key sensor of metabolic stress that converts systemic signals into a local, pro-atherogenic cellular program. The RBP4-STRA6 signaling axis is thereby identified as a novel therapeutic target. Selectively inhibiting this non-classical pathway may provide a new strategy to uncouple metabolic disease from its destructive vascular consequences.

## 1 Introduction

### 1.1 Atherosclerosis: more than a lipid storage disease

For decades, the prevailing view of atherosclerosis centered on the passive accumulation of lipids within the arterial wall. While dyslipidemia is an undisputed and critical initiating factor, this “lipid-retention” model is insufficient to explain the complex, progressive nature of the disease (1). Modern evidence has recast atherosclerosis as a chronic, non-resolving inflammatory disease driven by a dynamic interplay between cellular dysfunction, oxidative stress, and maladaptive immune responses within the vessel wall (2). The vascular endothelium, a monolayer of cells forming the inner lining of blood vessels, is no longer seen as a simple structure but as a critical signaling and homeostatic organ. Endothelial dysfunction is a sentinel event in atherogenesis, which precedes and enables the subsequent pathological changes (3). This dysfunctional state is characterized by increased permeability, increased expression of leukocyte adhesion molecules, and a switch from an anti-thrombotic, vasodilatory phenotype to a pro-thrombotic, pro-inflammatory, and vasoconstrictive one, setting the stage for lesion formation.

### 1.2 The senescent endothelial cell: an active driver of vascular inflammation and dysfunction

Cellular senescence, a state of irreversible cell cycle arrest, has emerged as a fundamental mechanism of aging and a key contributor to a host of age-related pathologies, including atherosclerosis (4, 5). Far from being a passive or dormant state, senescent cells are metabolically active and exhibit distinct morphological features, including an enlarged, flattened shape with increased cytoplasmic volume and vacuolization (6). These cells act as pathogenic hubs, characterized by profound changes in gene expression and resistance to apoptosis (6). The clinical relevance of this process to vascular disease is underscored by the identification of senescent ECs within human atherosclerotic plaques (7). These cells have been identified using histological and molecular assays for senescence-associated β-galactosidase (SA-β-gal) activity at pH 6.0, and elevated nuclear expression of the tumor suppressors p53 and the cyclin-dependent kinase inhibitor p21, confirmed via immunohistochemistry or immunofluorescence (8).

The accumulation of these senescent ECs is not merely a consequence of the disease but a primary causal contributor to its progression. The senescent phenotype directly fosters endothelial dysfunction by impairing the production of the master vasodilator, nitric oxide (NO), and promoting a prothrombotic and vasoconstrictive environment (4, 9). This functional decay cripples the ability of the endothelium to maintain vascular homeostasis, repair injury, and resist the formation of atherosclerotic lesions.

### 1.3 The senescence-associated secretory phenotype as a pathogenic amplifier

Perhaps the most deleterious feature of senescent cells is an acquired complex secretome known as the senescence-associated secretory phenotype (SASP) (10). The SASP consists of a potent cocktail of secreted factors, including pro-inflammatory cytokines such as Interleukin-1α (IL-1α), IL-1β, and IL-6; chemokines like monocyte chemoattractant protein-1 (MCP-1); various growth factors; and matrix-degrading proteases (11).

The SASP functions as a powerful pathogenic amplifier, transforming a localized cellular defect into a widespread tissue pathology. Through paracrine mechanisms, SASP factors can induce a state of senescence in neighboring healthy cells, spreading the dysfunctional phenotype throughout the endothelial monolayer (12). Furthermore, the chemokines within the SASP actively recruit inflammatory immune cells, such as monocytes and lymphocytes, from the circulation into the vessel wall, perpetuating a cycle of chronic, low-grade inflammation known as “inflammaging” (13). This self-sustaining inflammatory loop, orchestrated by the senescent ECs, is central to the chronic and progressive nature of atherosclerosis.

While diverse stimuli contribute to this inflammatory milieu, emerging evidence points to specific metabolic mediators as key instigators. Retinol-Binding Protein 4 (RBP4), primarily synthesized in the liver and adipose tissue, has surfaced as one such critical factor (14). While its physiological role is the mobilization of hepatic vitamin A stores, circulating RBP4 levels are pathologically elevated in metabolic syndromes, including obesity, type 2 diabetes, and cardiovascular disease (15). In these states, RBP4 transcends its carrier role, acting as an adipokine that induces systemic insulin resistance and vascular inflammation. This review focuses on the interaction between this pathogenic excess of RBP4 and endothelial STRA6 (16). Under physiological conditions, the majority of circulating holo-RBP4 is bound to transthyretin (TTR), forming a ternary complex that prevents renal filtration. TTR strictly regulates the bioavailability of RBP4; by sterically hindering the interaction between RBP4 and STRA6, TTR effectively limits the activation of downstream signaling pathways (17, 18). However, in metabolic disorders, the stoichiometric balance is often disrupted, leading to an increase in RBP4 not bound to TTR (free RBP4).

Crucially, this elevation of ‘Free RBP4’ is not solely a product of absolute RBP4 upregulation. In metabolic syndrome, a decrease in circulating TTR levels (acting as a negative acute-phase reactant) or post-translational modifications of RBP4 (e.g., C-terminal truncation) can reduce RBP4-TTR affinity, thereby increasing the fraction of bioactive, signaling-competent RBP4 accessible to endothelial STRA6 (19).We propose that this ‘uncoupled’ free RBP4 is the primary driver of the potent, non-classical JAK/STAT signaling observed in endothelial dysfunction (20).

While established triggers of EC senescence, such as replicative exhaustion, oxidative stress, and genotoxic damage, are well-documented, a critical knowledge gap remains in understanding the specific upstream signals that initiate this pro-atherogenic program, particularly in the context of systemic metabolic disease (21). Conditions such as obesity and type 2 diabetes are major accelerators of atherosclerosis, yet the molecular bridge linking systemic metabolic dysregulation to localized vascular cell senescence is not fully elucidated.

This review posits that the retinol-binding protein 4 (RBP4)-STRA6 axis serves as this critical link. Elevated circulating levels of RBP4 are a well-established hallmark of insulin resistance and obesity (22). We propose that in the vascular endothelium, RBP4 acts as a pathological, cytokine-like ligand that engages its cell surface receptor, STRA6. Crucially, the central argument of this review is that the primary pathogenic consequence of this interaction is not the facilitation of vitamin A transport—the canonical function of STRA6—but the direct activation of a non-classical, retinol-independent signaling cascade. This signaling pathway hijacks the core molecular machinery of cellular senescence, providing a direct and potent mechanism through which systemic metabolic stress drives the initiation and progression of atherosclerotic disease.

## 2 The dual identity of STRA6: from vitamin transporter to signaling hub

**Figure 1:**
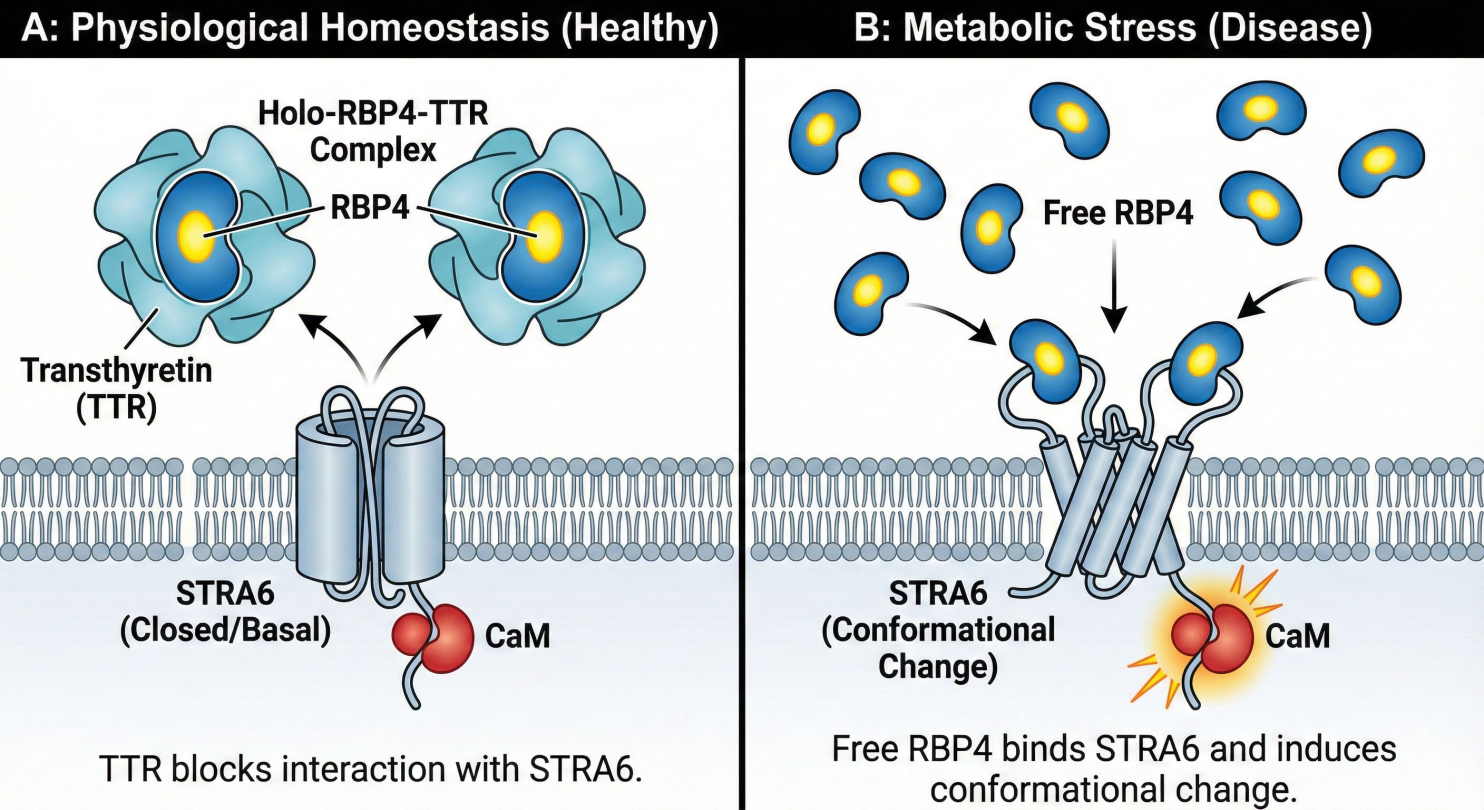
The dual functions of the STRA6 receptor. **(A)** Under homeostatic conditions, TTR binds Holo-RBP4 to prevent signaling, while STRA6 facilitates retinol uptake from Holo-RBP4, driven by intracellular coupling with CRBP-I and LRAT. Note the constitutive association of Calmodulin (CaM) with the receptor’s C-terminal domain. **(B)** Non-Classical Signaling (Metabolic Stress State): Pathogenic RBP4 ligands trigger the phosphorylation of the specific Y644 motif on the C-terminal tail. This recruits JAK2 to activate STAT transcription factors, driving the expression of genes (e.g., SOCS3) that promote inflammation and endothelial senescence. Abbreviations: RBP4, Retinol-Binding Protein 4; CaM, Calmodulin; JAK2, Janus Kinase 2; STAT, Signal Transducer and Activator of Transcription.

The protein Stimulated by Retinoic Acid 6 (STRA6) presents a fascinating case of molecular moonlighting, possessing two distinct and functionally separable identities. Initially characterized as an essential transporter for vitamin A, it has more recently been unveiled as a sophisticated signaling receptor. This dual functionality is not merely a biological curiosity but is central to understanding its tissue-specific roles in both health and disease (**Figure 1**, **Table 1**).

**Table 1:** Comparative analysis of the dual functions of STRA6.

Feature	Canonical transport function	Non-classical signaling function	References
Primary Ligand	Holo-RBP4 (as a source of retinol)	Free RBP4 (uncoupled from TTR)	(19)
Key Extracellular Interactor	Retinol-Binding Protein 4 (RBP4)	Retinol-Binding Protein 4 (RBP4)	(21)
Key Intracellular Interactors	Cellular Retinol-Binding Protein I (CRBP-I), Lecithin: Retinol Acyltransferase (LRAT)	Janus Kinase 2 (JAK2), Signal Transducer and Activator of Transcription 3/5 (STAT3/5)	(23, 24)
Primary Mechanism	Ligand binding, retinol extraction, and facilitated translocation across the plasma membrane	Ligand-induced receptor phosphorylation, recruitment of kinases, and activation of an intracellular signaling cascade	(18, 22)
Downstream Effector	Intracellular retinol and its metabolites (e.g., retinoic acid, retinyl esters)	Phosphorylated STAT dimers acting as nuclear transcription factors	(25, 26)
Primary Biological Outcome	Maintenance of cellular and tissue vitamin A homeostasis	Direct regulation of target gene expression (e.g., *SOCS3*, pro-inflammatory genes)	(4, 27)
Physiological Context	Essential for vision; contributes to systemic vitamin A uptake	Mediates metabolic crosstalk (e.g., insulin resistance); drives pathological responses in vascular tissues	(4, 28)

### 2.1 The canonical function: a retinol gatekeeper

The classical function of STRA6 is to mediate the cellular uptake of vitamin A from the holo-RBP4 complex (29). While the transmembrane topology of STRA6 was historically debated, with earlier models suggesting 11 transmembrane domains, recent cryo-electron microscopy (cryo-EM) studies have definitively resolved this structure. These studies reveal that STRA6 functions not as a passive pore, but as a dynamic homodimer with nine transmembrane helices per protomer (29). Crucially, this structural analysis identified Calmodulin (CaM) as an integral subunit constitutively bound to the STRA6 intracellular domain (23). Mechanistically, STRA6 facilitates the diffusion of retinol through a ‘lateral window’ directly into the cell membrane lipid bilayer rather than acting as a simple channel (29). This transport mechanism is tightly coupled to intracellular acceptors to drive directionality (25). Upon entering the cytosolic face, retinol is rapidly bound by Cellular Retinol-Binding Protein I (CRBP-I) and esterified by Lecithin: Retinol Acyltransferase (LRAT) (30). This enzymatic conversion creates a chemical “sink” that maintains the inward concentration gradient necessary for continuous uptake, effectively trapping vitamin A inside the cell (25).

The physiological necessity of this transport function is most profoundly demonstrated in the eye. The retinal pigment epithelium expresses exceptionally high levels of STRA6, and its transport activity is indispensable for supplying the retina with the vitamin A required for the visual cycle (31). Consequently, genetic ablation of Stra6 in mice leads to severe ocular vitamin A deficiency and associated visual impairments, even when systemic vitamin A levels are normal (4). This phenotype is mirrored in humans with loss-of-function mutations in the STRA6 gene, which cause Matthew-Wood syndrome, a severe developmental disorder characterized by microphthalmia or anophthalmia (small or absent eyes) and other systemic defects (32).

### 2.2 The non-classical paradigm: a cytokine-like receptor

Alongside its role as a transporter, a compelling body of evidence has established STRA6 as a cell surface signaling receptor, placing it in an emerging class of proteins known as “transceptors” or “cytokine signaling transporters” (33). This non-classical function fundamentally repositions STRA6 from a simple gatekeeper to an active transducer of extracellular information.

The initial clue to this signaling capacity came from the structural analysis of STRA6’s long intracellular C-terminal domain, which was found to contain a consensus phosphotyrosine motif (YxxL/V) that serves as a docking site for SH2 domain-containing proteins, a hallmark of receptors that activate the Janus Kinase/Signal Transducer and Activator of Transcription (JAK/STAT) pathway (18).

Subsequent experimental work confirmed this hypothesis, demonstrating that the binding of RBP4 to STRA6-expressing cells triggers the phosphorylation of a key tyrosine residue within this motif (18). While foundational studies identified the holo-RBP complex as the primary physiological agonist, the essential prerequisite for signaling is a specific ligand-induced conformational change in the receptor (18). This event initiates a canonical cytokine signaling cascade: the phosphorylated STRA6 recruits and activates JAK2, which in turn phosphorylates and activates the transcription factors STAT3 and STAT5. This receptor activation model suggests that STRA6 serves as a specific sensor for RBP4 conformations associated with nutrient or metabolic status (18).

This mode of action is entirely distinct from the classical genomic effects of vitamin A, which require retinol to be metabolized into retinoic acid to activate nuclear receptors (34). The STRA6 pathway thus represents a novel, non-canonical mechanism by which the vitamin A transport system can directly and rapidly regulate gene expression.

The structural integrity of STRA6’s transmembrane domains is critical for its transport function, but it is the cytosolic C-terminal domain that dictates its signaling competency (29). While recent cryo-EM studies confirm a conserved nine-transmembrane helix topology for the canonical receptor, alternative splicing events can generate isoforms with distinct intracellular variations (35, 36). Specifically, the retention or loss of the specific C-terminal region containing the Y644 motif acts as a molecular switch. Full-length isoforms possess the requisite docking site for JAK2, thereby coupling ligand binding to the STAT3/5 inflammatory cascade (18). In contrast, truncated isoforms lacking this cytosolic extension function exclusively as transporters, structurally incapable of engaging the JAK/STAT pathway (36). We postulate that the ratio of these isoforms on the endothelial surface is a determinant of vascular susceptibility to RBP4-induced senescence. A shift towards the expression of signaling-competent, full-length STRA6 in metabolic disease would explain the selective activation of endothelial inflammation despite systemic elevations in RBP4.

### 2.3 Mechanistic decoupling of transport and signaling: resolving the interdependence debate

A central question in the study of STRA6 is the relationship between its two functions: are they mechanistically coupled or independent? Initial *in vitro* studies suggested a tight interdependence, proposing that retinol transport was a prerequisite for signaling (22). However, this model is challenged by *in vivo* evidence from Stra6-null mice, which exhibit a dissociation between transport defects (ocular) and signaling phenotypes (insulin resistance) (4). The “transceptor” model reconciles these findings, positing that the initial ligand-induced conformational change is sufficient to trigger signaling, independent of the full transport cycle (37). This is supported by the distinct physiological context of the endothelium. Unlike the retinal pigment epithelium, where STRA6 is indispensable for vitamin A uptake, vascular endothelial cells can acquire retinoids via alternative, STRA6-independent mechanisms, such as passive diffusion or uptake from chylomicrons and lipoproteins (38, 39).

This physiological redundancy liberates endothelial STRA6 from the obligate burden of nutrient transport. Consequently, in the vasculature, STRA6 has been evolutionarily co-opted to function primarily as a metabolic sensor. Its activity is gated by the interaction between RBP4 and Transthyretin (TTR). Under homeostatic conditions, TTR sterically hinders RBP4 from binding STRA6 (17). However, in metabolic disorders, the stoichiometric balance is disrupted, generating a pool of “free” RBP4 (40). We propose that STRA6 detects this ‘uncoupled’ RBP4 as a distress signal, translating systemic metabolic status into immediate cellular action, bypassing the slower process of retinol metabolism. Furthermore, the downstream consequences of retinol uptake depend on its intracellular esterification. In activated endothelial cells, the saturation of intracellular retinoid-binding proteins may limit further transport, thereby favoring the stabilization of the RBP4-STRA6 signaling complex on the cell surface (41).

## 3 The RBP4-STRA6 signaling axis: a pro-senescent cascade in endothelial cells

### 3.1 A convergence of lipotoxicity and inflammation

The engagement of STRA6 by its ligand initiates a cascade that is far more complex than a simple cytokine interaction (42). In the context of metabolic syndrome, we propose the primary pathogenic ligand is “Free RBP4”—specifically, holo-RBP4 that exceeds the binding capacity of Transthyretin (TTR). This “uncoupled” state exposes the STRA6-binding interface that is otherwise sterically hindered by TTR. While the canonical pathway requires retinol-loaded (holo) RBP4, emerging evidence suggests that under metabolic stress, RBP4 may also act as a carrier for fatty acids, a state that potentially mimics the signaling-competent conformation. Crucially, the dissociation from TTR allows free RBP4 to induce a distinct conformational change in the STRA6 homodimer. Unlike the transient engagement seen in the transport cycle, this interaction stabilizes the receptor for sustained signaling (43).

A critical amplifier of this process is the dynamic upregulation of the receptor itself. While basal STRA6 expression in endothelial cells is lower than in the eye, pathological stimuli characteristic of atherosclerotic plaques—such as hypoxia and TNF-α—have been implicated in increasing its surface density (44). This creates a “vicious cycle”: metabolic stress elevates the pathogenic ligand (fatty acid-loaded, free RBP4), while local vascular inflammation upregulates the receptor, sensitizing specific vascular beds to these systemic signals (**Table 2**).

**Table 2:** Molecular components of the STRA6-induced pro-senescent microenvironment.

Component category	Key molecules	Proposed function in STRA6-mediated senescence	References
Upstream Signal	RBP4, STRA6	Ligand-receptor complex that initiates the signaling cascade in response to systemic metabolic stress.	(16, 45)
Primary Transducers	JAK2, STAT3, STAT5	Kinase and transcription factors that translate the extracellular signal into a nuclear transcriptional response.	(24, 46)
Inflammatory Amplifiers	NLRP3, ASC, Caspase-1	Components of the inflammasome complex that sense cellular stress (ROS) and process pro-inflammatory cytokines.	(47, 48)
SASP Effectors	IL-1β, IL-18 (Unconventional); IL-6, MMPs (Classical)	IL-1β/IL-18 are released via Gasdermin D pores; IL-6/MMPs via classical secretion. They actively recruit monocytes and induce paracrine senescence.	(12, 49)
Senescence Executioners	ATM, γH2AX, p53, p21	Key players in the DNA Damage Response and cell cycle control that mediate irreversible growth arrest.	(50, 51)

### 3.2 Primary signal transduction: activation of the JAK/STAT pathway

The transduction of this signal is strictly governed by the genetic identity and structural state of the receptor. As established in the structural analysis, only the full-length isoform containing the cytosolic Y644 motif can recruit JAK2 (18, 52). Furthermore, it is crucial to note the spatial proximity of this JAK2 docking site to the constitutively bound Calmodulin (CaM) subunit on the C-terminus (23, 53). This structural arrangement suggests that the phosphorylation of Y644 and the subsequent recruitment of STAT3/5 might be gated by intracellular calcium fluxes. Specifically, calcium binding to CaM likely induces a conformational shift in the receptor’s C-terminus, thereby exposing the Y644 motif to JAK2 and facilitating the initiation of the signaling cascade. This points to a potential “coincidence detection” mechanism, where metabolic stress and calcium dysregulation synergize to launch the inflammatory program (54).

In the context of vascular biology, the JAK/STAT pathway is a central mediator of inflammation and cellular stress responses (28). Key transcriptional targets of STRA6-activated STATs include Suppressor of Cytokine Signaling 3 (SOCS3), a protein that induces insulin resistance by inhibiting the insulin receptor signaling cascade, providing a direct link between RBP4-STRA6 signaling and metabolic dysfunction (55).

**Figure 2:**
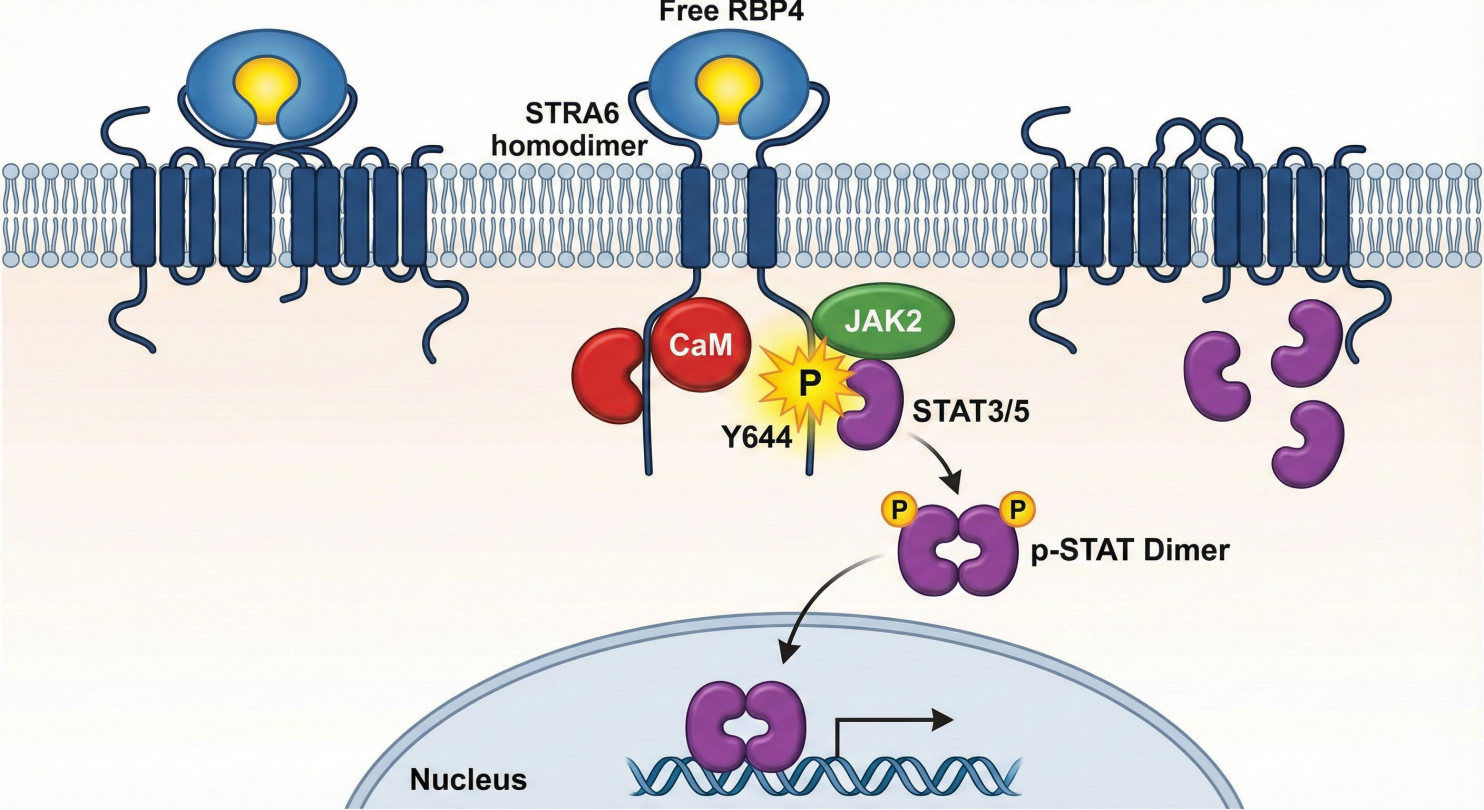
Activation of the JAK/STAT signaling cascade by the RBP4-STRA6 axis. This figure details the initial signal transduction event following ligand engagement. The binding of free RBP4 (stoichiometrically uncoupled from TTR) to the STRA6 receptor constitutes the pathogenic trigger. This engagement induces a structural shift that activates the associated JAK2, which phosphorylates key tyrosine residues (p-Tyrosine) on the intracellular C-terminal domain of STRA6. These phosphotyrosine sites serve as docking platforms for latent cytoplasmic STAT proteins. Once recruited, STATs are phosphorylated by JAK2 (p-STAT), leading to their dissociation from the receptor and the formation of stable p-STAT dimers. These activated dimers then translocate into the nucleus to regulate the transcription of target genes. RBP4, Retinol-Binding Protein 4; STRA6, Stimulated by Retinoic Acid 6; JAK2, Janus Kinase 2; STAT, Signal Transducer and Activator of Transcription; p-STAT, phosphorylated STAT.

While SOCS3 acts as a classical feedback inhibitor to dampen JAK/STAT signaling, its overexpression in this context paradoxically fuels pathology (56, 57). Instead of effectively silencing the STRA6 axis, sustained SOCS3 accumulation preferentially inhibits the insulin receptor substrate (IRS), thereby entrenching insulin resistance while leaving the STRA6-NLRP3 inflammatory loop active (45). Furthermore, STATs are known to regulate the expression of a wide array of genes involved in inflammation, cell adhesion, lipid metabolism, and proliferation, priming the endothelial cell for a pro-atherogenic response (**Figure 2**).

Importantly, this transcriptional priming is likely reinforced by a parallel pathway. RBP4 has been shown to directly activate Toll-like Receptor 4 (TLR4), a canonical driver of NF-κB signaling (15). We suggest a synergistic model where STRA6-mediated STAT activation and TLR4-mediated NF-κB activation converge to maximally upregulate inflammasome components, rendering endothelial cells hypersensitive to secondary stress signals (24). Besides STRA6 and TLR4, RBP4 interaction with the receptor PIRB (Paired Immunoglobulin-like Receptor B) and RBPR2 has been documented in other tissues. While PIRB is known to mediate RBP4-induced insulin resistance in myeloid cells, its expression and functional relevance in the vascular endothelium appear negligible compared to the robust STRA6-JAK/STAT axis, positioning STRA6 as the dominant endothelial sensor for RBP4 (46).

### 3.3 Fueling the fire: crosstalk with the NLRP3 inflammasome and SASP amplification

While JAK/STAT activation provides the initial pro-inflammatory signal, a crucial amplification step is required to establish the robust and self-sustaining inflammatory microenvironment characteristic of cellular senescence. This review proposes that the STRA6-JAK/STAT axis achieves this by engaging the NLRP3 inflammasome, a multiprotein complex of the innate immune system that acts as a central hub for processing pro-inflammatory cytokines (58).

The activation of the NLRP3 inflammasome is a tightly regulated two-step process.

Signal 1 (Priming): The first step involves the transcriptional upregulation of key inflammasome components. The STAT transcription factors, activated by the RBP4-STRA6-JAK2 axis, are proposed to drive the expression of NLRP3 (the gene encoding the sensor protein) and IL1B (the gene encoding the cytokine precursor pro-IL-1β).

Signal 2 (Activation): The second step triggers the assembly of the inflammasome complex. A definitive activator of NLRP3 is the elevation of intracellular reactive oxygen species (ROS). The STRA6-JAK2 axis stimulates NADPH oxidase (Nox) activity, likely through the phosphorylation of regulatory subunits such as p47phox or by facilitating the assembly of the Nox complex, generating a rapid ROS surge (26).

Furthermore, considering that mitochondrial dysfunction is a hallmark of metabolic stress, we posit that STRA6 signaling may synergize with mitochondrial ROS leakage, creating a dual-source oxidative stress that significantly lowers the threshold for NLRP3 assembly (59). This oxidative stress acts as the requisite second signal, inducing the oligomerization of the NLRP3 sensor protein and the subsequent recruitment of the adaptor protein ASC.

Once activated, NLRP3 recruits the adaptor protein ASC (Apoptosis-associated speck-like protein containing a CARD), which in turn recruits pro-Caspase-1. The proximity of multiple pro-Caspase-1 molecules facilitates their auto-cleavage and activation (27). Active Caspase-1 is a protease whose primary substrates are the inactive cytokine precursors, pro-IL-1β and pro-IL-18. Caspase-1 cleaves these precursors into their mature, biologically active forms. Simultaneously, Caspase-1 cleaves Gasdermin D, generating N-terminal fragments that oligomerize to form pores in the plasma membrane.

Crucially, in the context of senescence, this pore formation is regulated to a sub-lytic level. This allows for the sustained, unconventional secretion of mature IL-1β without triggering immediate cell death (pyroptosis), thereby preserving the metabolically active senescent state (47). These pores facilitate the rapid, unconventional secretion of mature IL-1β into the extracellular space, defining the maturation of the SASP (48).

**Figure 3:**
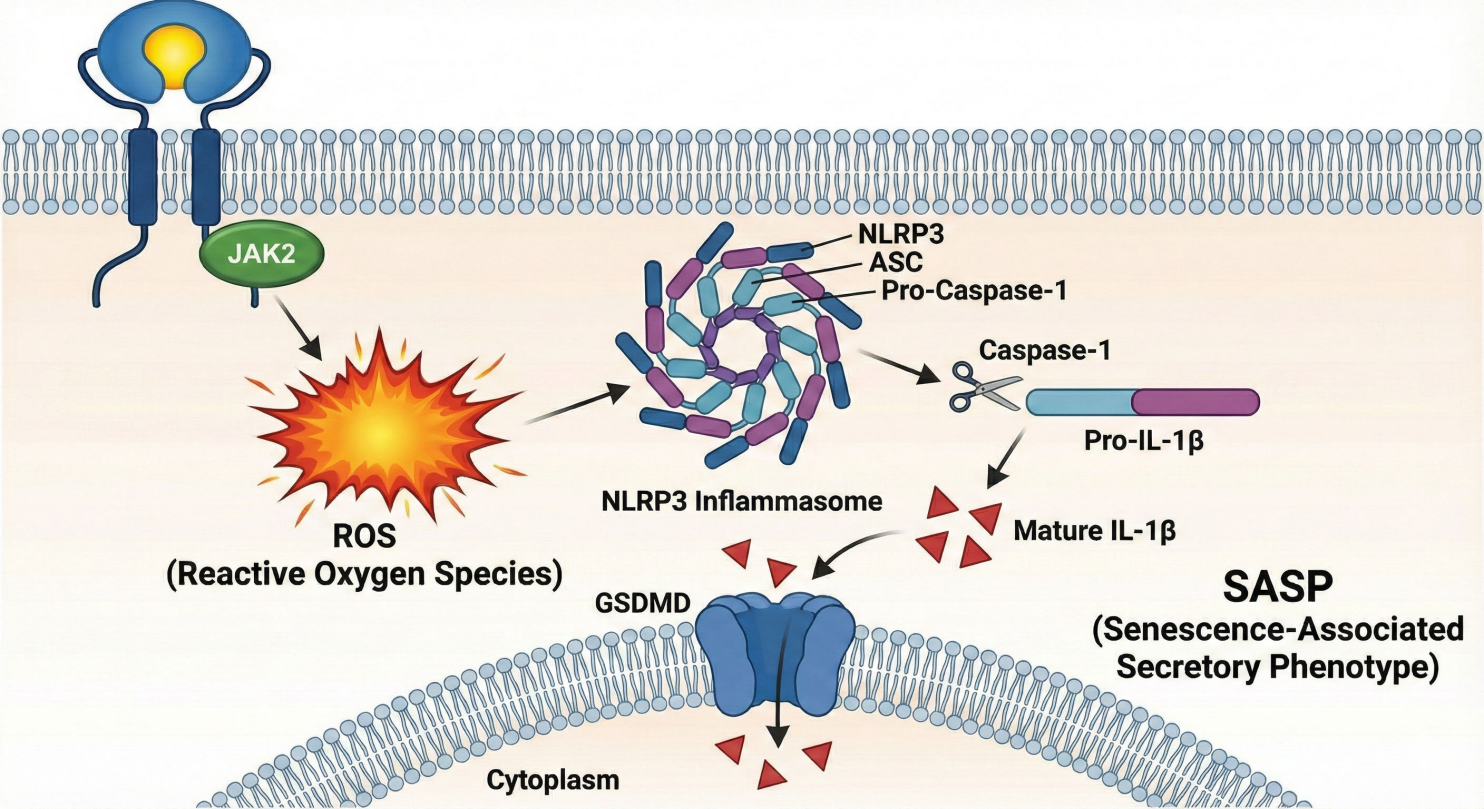
Amplification of the pro-senescent signal through crosstalk with the NLRP3 inflammasome. The initial signal from the RBP4-STRA6 axis is amplified via a two-signal mechanism for NLRP3 inflammasome activation. Signal 1 (Priming): STAT dimers, previously activated by the STRA6-JAK pathway, translocate to the nucleus and drive the transcriptional upregulation of key inflammasome components, including NLRP3 and pro-IL-1β. Signal 2 (Activation): The STRA6-JAK pathway also stimulates the production of intracellular reactive oxygen species (ROS), which serves as the trigger for the assembly of the NLRP3 inflammasome complex (NLRP3, ASC, and pro-Caspase-1). The assembled inflammasome facilitates the auto-activation of Caspase-1, which then cleaves pro-IL-1β into its mature, biologically active form, IL-1β. Secreted IL-1β is a key effector of the senescence-associated secretory phenotype (SASP), propagating inflammation and senescence. STAT, Signal Transducer and Activator of Transcription; NLRP3, NOD-like receptor family pyrin domain containing 3; IL-1β, Interleukin-1β; ROS, Reactive Oxygen Species; ASC, Apoptosis-associated speck-like protein containing a CARD; SASP, Senescence-Associated Secretory Phenotype.

The secretion of mature IL-1β is a defining event in the maturation of the SASP. IL-1β is a potent pro-inflammatory and pro-senescent cytokine that can act in an autocrine and paracrine fashion, binding to its receptor (IL-1R) on the senescent cell and its neighbors to further amplify inflammatory signaling and induce senescence in adjacent cells (49). This creates a powerful and pathogenic feed-forward loop, where an initial metabolic signal from RBP4 is translated by STRA6 into a self-propagating wave of inflammation and senescence along the endothelial lining (**Figure 3**).

### 3.4 Convergence on core senescence machinery: the DNA damage response and p53 activation

The final stage in the execution of the senescence program involves the convergence of the upstream inflammatory signals onto the core machinery that governs cell cycle control and genome integrity. The chronic, low-grade inflammatory and oxidative microenvironment created by the STRA6-JAK/STAT-NLRP3 axis is a potent source of cellular damage. The combination of elevated ROS production and the actions of SASP cytokines are well-established inducers of DNA damage, including single- and double-strand breaks (60).

Persistent sublethal damage triggers the DNA Damage Response (DDR) surveillance network. At the apex of this cascade is the kinase Ataxia-Telangiectasia Mutated (ATM). Upon detection of double-strand breaks, ATM phosphorylates the histone variant H2AX at Serine 139 (γH2AX), establishing foci that recruit DNA repair complexes (61). Under chronic RBP4-STRA6 signaling, this sustained ATM activation stabilizes p53, thereby shifting the cellular fate from canonical DNA repair mechanisms to permanent senescence (50).

If the damage is irreparable or the DDR signal is sustained over time, the pathway pivots from repair to the induction of a permanent cell fate program, either apoptosis or senescence. In the context of the chronic, low-level stress induced by the STRA6 cascade, the outcome is preferentially senescence. Unlike acute, high-intensity damage which typically triggers apoptosis, persistent but sublethal stress signals—such as those generated by metabolic inflammation—favor p53-mediated cell cycle arrest and senescence as a survival strategy to prevent the propagation of damaged genomes (62). A key effector of this decision is the tumor suppressor protein p53 (63). The sustained DDR signal leads to the stabilization and activation of p53, preventing its degradation and allowing it to accumulate in the nucleus (64).

As a master transcription factor, activated p53 drives the expression of a suite of genes that enforce the senescent state. Its most critical downstream target in this context is the cyclin-dependent kinase inhibitor p21 (CDKN1A) (51). p21 potently inhibits the activity of cyclin-CDK complexes (specifically Cyclin E-CDK2 and Cyclin A-CDK2), which are required for the G1/S and S/G2 transitions of the cell cycle. By inhibiting these kinases, p21 prevents the phosphorylation of the Retinoblastoma (Rb) protein, keeping it in its active, hypo-phosphorylated state where it sequesters E2F transcription factors and blocks the expression of genes required for DNA replication and cell division (65). This p53-p21-Rb pathway establishes the stable and irreversible cell cycle arrest that is the defining hallmark of cellular senescence.

While the p53-p21 axis primarily initiates this arrest, the subsequent upregulation of the cyclin-dependent kinase inhibitor p16INK4a likely serves to lock the cells in this state, preventing cell cycle re-entry even if the initial metabolic stress subsides (66, 67). Reinforcing this network, STRA6 signaling engages in crosstalk with the Wnt/β-catenin pathway. The sequestration of β-catenin by RBP4-activated STRA6 prevents its nuclear translocation, thereby suppressing pro-proliferative Wnt target genes and further solidifying the cell cycle arrest characteristic of the pro-atherogenic phenotype (53).

## 4 Pathophysiological integration and therapeutic horizons

### 4.1 A unified model: from metabolic stress to atherosclerotic plaque

The evidence and mechanisms detailed in this review can be synthesized into a unified, multi-scale model that directly links systemic metabolic disease to the initiation of atherosclerosis at the cellular level. The process begins with systemic metabolic dysfunction—driven by conditions like obesity, insulin resistance, and type 2 diabetes.

Driven by insulin resistance in adipose tissue, circulating RBP4 levels rise significantly. This elevated holo-RBP4 acts as a chronic, pathological signal that is sensed by the STRA6 receptor on the surface of vascular endothelial cells. The engagement of STRA6 triggers its non-classical, retinol-independent signaling function, activating the intracellular JAK/STAT pathway. This primary signal is then robustly amplified through crosstalk with the NLRP3 inflammasome, which is primed by STAT-mediated transcription and activated by JAK-induced ROS production. The resulting activation of Caspase-1 leads to the maturation and secretion of a potent SASP, rich in IL-1β and other pro-inflammatory mediators.

**Figure 4:**
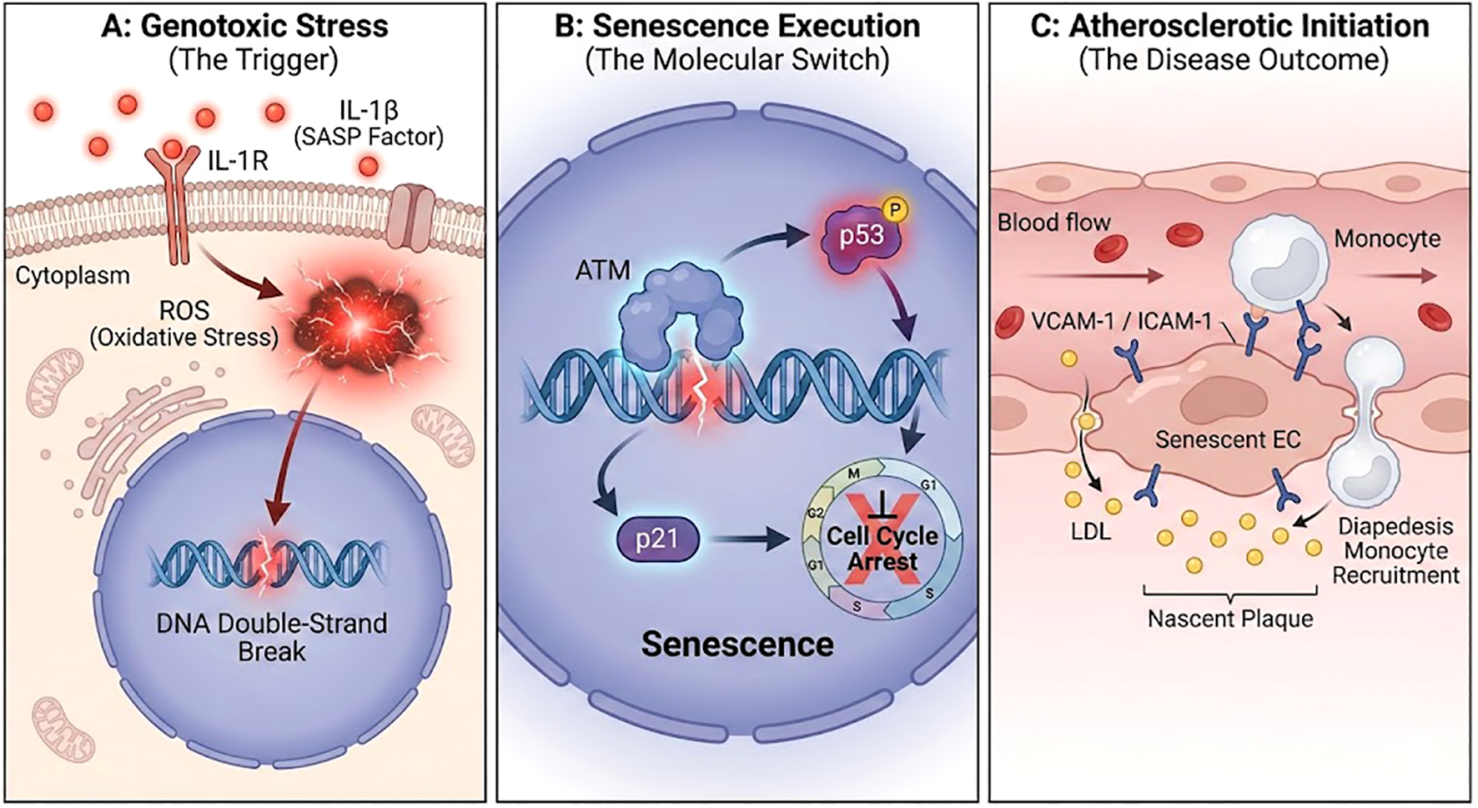
A unified model linking the STRA6-induced pro-inflammatory microenvironment to endothelial senescence and the initiation of atherosclerosis. This multi-panel diagram summarizes the final stages of the pro-atherogenic cascade. **(A)** Genotoxic Stress: The chronic microenvironment acts on the endothelial cell. Secreted SASP factors (e.g., IL-1β) bind to their cognate membrane receptors (IL-1R), causing a sustained increase in intracellular Reactive Oxygen Species (ROS). This oxidative stress induces persistent DNA damage, such as double-strand breaks, within the nucleus. **(B)** Senescence Execution: This genotoxic stress activates the DNA Damage Response (DDR), orchestrated by kinases like ATM, which in turn stabilizes and activates p53. Activated p53 drives the expression of cell cycle inhibitors (e.g., p21), leading to irreversible cell cycle arrest and the establishment of the senescent phenotype. **(C)** Initiation of Atherosclerotic Lesion Formation: Senescent endothelial cells contribute to atherogenesis by increasing vascular permeability to LDL cholesterol and upregulating surface adhesion molecules (VCAM-1/ICAM-1), which mediate the recruitment of circulating monocytes into the subendothelial space. This process marks the formation of an early atherosclerotic lesion. Abbreviations: SASP, Senescence-Associated Secretory Phenotype; ROS, Reactive Oxygen Species; ATM, Ataxia-Telangiectasia Mutated; LDL, Low-Density Lipoprotein.

The DDR culminates in the activation of the p53-p21 axis, enforcing an irreversible cell cycle arrest and locking the endothelial cell into a senescent state. The accumulation of these senescent ECs, coupled with their deleterious SASP, fundamentally compromises the integrity of the vascular endothelium. This leads to the cardinal features of endothelial dysfunction: compromised barrier function — mediated by the SASP-induced disruption of adherens junctions and downregulation of proteins like VE-Cadherin — increasing permeability to lipoproteins (LDL), and the upregulated surface expression of VCAM-1 and ICAM-1. These adhesion molecules mediate the tethering and diapedesis of circulating monocytes into the subendothelial space. These events collectively facilitate the infiltration of lipids and immune cells into the subendothelial space, marking the formation of the nascent fatty streak and initiating the inexorable progression of the atherosclerotic plaque (**Figure 4**).

### 4.2 Therapeutic implications: targeting STRA6 signaling

This refined understanding of STRA6’s role in vascular pathology opens new and exciting therapeutic avenues. The central premise for intervention is the selective inhibition of STRA6’s pro-senescent signaling function while ideally preserving its canonical transport function, which remains important for vitamin A homeostasis in other tissues, particularly the eye.

Stabilizing the TTR-RBP4 Complex (Ligand Sequestration): Since the dissociation of RBP4 from Transthyretin (TTR) is a prerequisite for signaling activation, a novel upstream strategy is to target the ligand itself (17, 39). Small molecule kinetic stabilizers, similar to tafamidis used in TTR amyloidosis, could be engineered to reinforce the RBP4-TTR interaction. By sequestering RBP4 within this high-affinity complex, the “free” RBP4 pool responsible for activating endothelial STRA6 would be depleted, effectively silencing the metabolic distress signal without interfering with total vitamin A circulation (68–70).

Interestingly, agents that lower serum RBP4 levels, such as Fenretinide, have shown promise in improving insulin sensitivity in clinical trials. Our model suggests that the cardiovascular benefits of such therapies may stem specifically from the reduced occupancy of endothelial STRA6, validating this axis as a druggable target. Beyond kinetic stabilization, future strategies could also explore gene therapies aimed at increasing endogenous TTR expression to match RBP4 overexpression, or approaches to accelerate the clearance of circulating free RBP4 (71).

Isoform-Specific Modulation (Splicing Switching): Our “Signaling Dominance” hypothesis highlights that only the full-length STRA6 isoform drives senescence. This opens the door for RNA-based therapies, such as Antisense Oligonucleotides (ASOs), designed to induce exon skipping or modulate splicing factors (72–74). The goal would be to force a “molecular switch” in endothelial cells: shifting expression away from the signaling-competent full-length receptor towards the truncated, transport-only isoform (75, 76). This strategy offers the highest degree of precision, selectively ablating the pathological inflammatory function while leaving the vitamin A transport machinery intact (33).

This specificity offers a distinct advantage over targeting broad-spectrum pattern recognition receptors like TLR4. Targeting the STRA6 axis specifically uncouples the metabolic trigger (RBP4) from vascular inflammation without compromising the host’s generalized immune defense mechanisms (4). While this approach does not resolve the systemic overexpression of RBP4, it effectively shields the vulnerable vascular endothelium from its pathogenic signaling.

Interdicting Downstream Amplification: For immediate clinical translation, repurposing existing drugs that target the downstream effector nodes remains a viable parallel strategy (77). JAK inhibitors (e.g., Ruxolitinib) can block the initial signal transduction, while emerging NLRP3 inflammasome inhibitors can sever the self-sustaining amplification loop (78). These agents could serve as a “brake” on the established senescent phenotype, complementing the upstream precision strategies described above.

Safety Considerations and Potential Risks: Despite the therapeutic promise, targeting this axis requires a careful consideration of safety profiles. Systemic inhibition of STRA6 must be designed to avoid interfering with ocular vitamin A uptake to prevent visual toxicity. Furthermore, regarding the inhibition of downstream targets, clinical caution is also warranted. JAK inhibitors (e.g., Tofacitinib, Ruxolitinib) carry “black box” warnings for increased risks of thrombosis and major adverse cardiovascular events (MACE) in certain populations, presenting a paradox where the treatment for vascular senescence might exacerbate thrombotic risk (79). Similarly, long-term blockade of the NLRP3 inflammasome raises concerns about immunosuppression and susceptibility to infections. Therefore, future therapeutic strategies might favor endothelium-specific delivery systems or allosteric modulators of STRA6 that selectively decouple the signaling arm without compromising systemic immunity or ocular nutrition (80).

### 4.3 Future directions: validating the model and uncovering nuances

While the proposed model provides a compelling framework, rigorous experimental validation is essential to translate these concepts into clinical reality. Several key research avenues must be pursued:

Genetic Models for Functional Uncoupling: The most definitive way to dissect the roles of STRA6’s dual functions *in vivo* is through the creation of sophisticated genetic models. The development of knock-in mice expressing a signaling-deficient but transport-competent version of STRA6—for instance, through a point mutation at the critical Y644 phosphorylation site (Y644F)—is a critical next step (81). Crossing these mice onto an atherosclerosis-prone background (e.g., Apoe−/− or Ldlr−/−) and challenging them with high-fat diets or exogenous RBP4 would allow for the unambiguous determination of the specific contribution of STRA6 signaling to endothelial senescence and lesion development.

Proteomic Characterization of the STRA6-Induced SASP: While the regulatory role of IL-1β is a central tenet of this model, the full composition of the SASP driven by STRA6 activation remains to be mapped. Unbiased, mass spectrometry-based proteomic analysis of the secretome from ECs stimulated with holo-RBP4 is required (82). This approach will not only validate the upregulation of inflammasome-dependent cytokines but is poised to identify novel, STRA6-specific SASP biomarkers distinct from classical senescence (83).

Preclinical Pharmacological Studies: The therapeutic potential of targeting downstream nodes must be tested in relevant animal models. Atherosclerosis-prone mice with diet-induced metabolic syndrome could be treated with specific JAK2 inhibitors or NLRP3 inflammasome inhibitors to assess whether these interventions can prevent or reverse RBP4-driven vascular senescence, endothelial dysfunction, and plaque progression.

Translational Human Studies: The relevance of this pathway in human disease needs to be established. This can be achieved through large-scale cohort studies investigating the association between plasma RBP4 levels, functional genetic polymorphisms in the STRA6 gene (84), and validated biomarkers of endothelial senescence, subclinical atherosclerosis (e.g., carotid intima-media thickness), and future cardiovascular events. Furthermore, validating the “Signaling Dominance” hypothesis in humans is critical. Future studies should aim to quantify the ratio of full-length versus truncated STRA6 isoforms in human atherosclerotic plaques compared to healthy endothelium to confirm their differential expression in disease states.

The RBP4-STRA6-senescence axis may represent a final common pathway through which diverse metabolic risk factors converge to inflict damage upon the vasculature. This elevates its potential importance from one of many inflammatory pathways to a central, integrating hub of metabolic-vascular pathology. If validated, it would become a high-priority target for the development of next-generation therapies aimed at preventing and treating atherosclerotic cardiovascular disease.

## 5 Conclusion

The conceptualization of STRA6 is shifting from merely a vitamin A transporter to a key pathogenic signaling receptor in the vascular endothelium. In metabolic disease, elevated Retinol-Binding Protein 4 (RBP4) acts as a systemic stress signal. The RBP4-STRA6 complex activates a non-classical, retinol-independent JAK/STAT pathway. This signal is amplified by the NLRP3 inflammasome, promoting a pro-inflammatory state that drives cellular senescence via the p53/p21 axis, ultimately contributing to atherosclerosis.

This redefines STRA6 as a crucial metabolic sensor. This new understanding opens therapeutic possibilities: selectively inhibiting the pathological signaling function of STRA6 could sever the link between metabolic and cardiovascular disease while preserving its essential transport functions.
